# The Role of Vitamin D in Stroke Prevention and the Effects of Its Supplementation for Post-Stroke Rehabilitation: A Narrative Review

**DOI:** 10.3390/nu14132761

**Published:** 2022-07-04

**Authors:** Klaudia Marek, Natalia Cichoń, Joanna Saluk-Bijak, Michał Bijak, Elżbieta Miller

**Affiliations:** 1 Department of Neurological Rehabilitation, Medical University of Lodz, Milionowa 14, 93-113 Lodz, Poland; elzbieta.dorota.miller@umed.lodz.pl; 2 Biohazard Prevention Center, Faculty of Biology and Environmental Protection, University of Lodz, Pomorska 141/143, 90-236 Lodz, Poland; natalia.cichon@biol.uni.lodz.pl (N.C.); michal.bijak@biol.uni.lodz.pl (M.B.); 3 Department of General Biochemistry, Faculty of Biology and Environmental Protection, University of Lodz, Pomorska 141/143, 90-236 Lodz, Poland; joanna.saluk@biol.uni.lodz.pl

**Keywords:** stroke, 25(OH)D deficiency, supplementation, vitamin D, rehabilitation

## Abstract

Hypovitaminosis D is a serious public health problem, representing an independent factor in mortality among the general population. Vitamin D deficiency may affect up to one billion people worldwide. Recently, the potential association between vitamin D levels and stroke has gained increasing attention. Many studies suggest that maintaining normal serum vitamin D levels is associated with improvement of the cardiovascular system and a reduction in stroke risk. As a neurosteroid, vitamin D influences brain development and function and immunomodulation and affects brain neuroplasticity. It supports many processes that maintain homeostasis in the body. As stroke is the second most common cause of death worldwide, more studies are needed to confirm the positive effects of vitamin D supplementation, its dosage at different stages of the disease, method of determination, and effect on stroke onset and recovery. Many studies on stroke survivors indicate that serum vitamin D levels only offer insignificant benefits and are not beneficial to recovery. This review article aims to highlight recent publications that have examined the potential of vitamin D supplementation to improve rehabilitation outcomes in stroke survivors. Particular attention has been paid to stroke prevention.

## 1. Introduction

Vitamin D was discovered in 1913 by McCollum and Davis [1]. According to The National Academy of Medicine, vitamin D deficiency occurs below a concentration of 20 ng/mL, while vitamin D insufficiency is a 25(OH)D concentration in the range of 21–29 ng/mL [2]. Vitamin D is fat soluble, and normal bone development and metabolism are its primary functions. It increases the absorption of calcium, magnesium and phosphate [3,4]. Scientists warn that vitamin D deficiency could affect up to one billion people worldwide [5]. Hypovitaminosis D is a serious public health problem, being an independent factor influencing mortality in the general population [6]. Vitamin D plays a significant role in the prevention and treatment of many diseases: autoimmune diseases, influenza, depression, heart disease, cancer, fractures, type 2 diabetes, and even central nervous system diseases including stroke [7,8,9,10,11]. Vitamin D can be produced in the skin as a result of exposure to sunlight.

Vitamin D is the precursor to the active form of 1,25-dihydroxyvitamin D and is found in two forms. Vitamin D2 (ergocalciferol) is formed by UV-B irradiation of plants, fungi, and yeast containing the steroid ergosterol. Vitamin D3 (cholecalciferol) is synthesized in human skin, where exposure to UV-B radiation converts 7-dehydrocholesterol to previtamin D3. However, there is a lack of consistency in the use of UV-B wavelengths in the scientific literature, where values between 270 and 320 nm are used [12,13,14,15].

Some foods are sources of vitamin D2 and D3: oily fish, fish liver oil (vit. D3), dietary supplements (vit. D2 and D3), and fortified foods, breakfast cereals, and margarines (vit. D2) [16]. In humans, most vitamin D is obtained by vitamin D3 production in the skin. However, vitamin D must be changed into its active form, 1,25-dihydroxyvitamin D3, to exert its full effects on the body [1].

Hydroxylation of vitamin D2 and D3 occurs in two stages. The first step occurs in the liver using several enzymes, the most important of which is *CYP2R1* (D3 25-hydroxylase). It results in the formation of an inactive 25(OH)D precursor (25-hydroxyvitamin D) [12,17,18]. The next step takes place in the kidneys, where the enzyme 1α-hydroxylase (*CYP27B1*) converts 25(OH)D to 1,25-dihydroxyvitamin D2 or D3 [17] (calcitriol: an important modulator of calcium and phosphate homeostasis). In addition to the kidneys, the activating enzyme *CYP27B1* and 1,25-dihydroxyvitamin D3 are also present in various tis-sues, including the human brain, cardiovascular system, pancreatic islets, and immune cells [19,20].

Lack of vitamin D leads to impaired absorption of nutrients. Adequate vitamin D concentrations increase calcium and phosphorus absorption by 30–40 and 80 percent, respectively; hence, in cases of deficiency, P absorption through the intestines is reduced to about 60 percent, and calcium to only 10–15 percent [21].

The aim of the review was to present studies on vitamin D supplementation in stroke patients, the efficacy of rehabilitation after vitamin D supplementation in stroke patients was investigated, and to review the results of research, the efficacy of supplementation, and the type, form and amount of administration of vitamin D to patients. The criteria for inclusion in the studies were compared, the duration of the ongoing studies and the scales used on the basis of which the researchers reached their conclusions are presented.

## 2. Methods

The MEDLINE and EMBASE databases were used for the literature search. A total number of 125 articles were analyzed, including 71 original research papers and 54 reviews (meta-analyses, systematic reviews, literature reviews). In this paper, we allowed articles mainly from the last 10 years to be included. Most explored articles focused on vitamin D supplementation in neurological patients, in this case after stroke, and were published in the English language. Search terms included vitamin D supplementation, post-stroke vitamin D supplementation, vitamin D action in CNS, vitamin D supplementation during rehabilitation and protective effect of vitamin D. Moreover, we excluded non-English articles. No restrictions were set for the type of stroke, stage of stroke and disability level. Independently, three authors searched the databases for articles on post-stroke supplementation of vitamin D.

## 3. Role of Vitamin D on Brain

Recent literature reports have highlighted the importance of vitamin D supplementation in ischemic stroke. Siniscalchi, Lochner, Anticoli et al. suggest that low vitamin D levels are more associated with ischemic stroke than hemorrhagic stroke and emphasize that the number of studies is insufficient [22]. The severity of the disease and its prognosis and outcomes worsen with vitamin D deficiency [23]. Detailed studies on the mechanism of nerve protection by which vitamin D relieves an eruption and influences complications following a stroke are lacking. This may be a limitation of brain endothelial permeability and the regulation of the transport of molecules to the central nervous system for which the blood–brain barrier (BBB) is responsible. Ischemia-related BBB diseases include increased production of reactive oxygen (ROS), nitric oxide, intracellular changes in calcium and the production of vascular endothelial growth factor (VEGF) [24]. However, data are limited and inconclusive, and as the efficacy of vitamin D on post-stroke rehabilitation outcomes and cardiovascular risk reduction is unknown, caution is advised when using vitamin D supplementation [25,26,27,28]. Studies have identified vitamin D in peripheral neurons and several cell types of the central nervous system [13,29]. The action of 1,25-dihydroxyvitamin D3 has been linked to the vitamin D receptor (VDR) response and to a variety of biological processes such as cell growth, differentiation and proliferation, and immune modulation. All of the above processes are involved in influencing brain function, which are associated with gene interactions because the VDR exerts its genomic effects through the nuclear transcription factor gene [30].

Polymorphisms of *CYP2R1*, *CYP27B1*, *CYP24A1* and *VDR* can influence vitamin D concentration in the body. *CYP27B1* encodes the process of 25(OH)D hydroxylation by 1 α-hydroxylase, generating 1,25-dihydroxy vitamin D. *CYP24A1* encodes a 24-hydroxylase enzyme capable of inactivating both 25(OH)D and 1,25(OH)D by hydroxylation [31]. After being secreted by the kidneys, 1,25(OH)2D is bound to VDBP and transported to the target organs, where VDR mediates its absorption [32]. VDR, encoded by the VDR gene, is a key receptor of the endocrine and autocrine vitamin D system. VDR is highly expressed in tissues of the small intestine, colon, bone, kidney, skin, vascular system, immune system, brain, muscle, and endocrine organs. Its participation in many different tissue and cell types indicates the likelihood of involvement in autoimmune, cancer, or cardiovascular disease pathology [33].

Calcitriol, the active form of vitamin D, affects gene expression, both directly and indirectly through the nuclear vitamin D receptor (VDR) [34]. Compound 1,25(OH)2D3 is a potent modulator of the immune system. As well as regulating inflammation, it also affects cell cycle control, neuromuscular function, and neuroprotection by controlling neurotrophin production [35]. The enzyme 1α-hydroxylase and vitamin D metabolic pathways have been observed in the human brain and in cerebrospinal fluid [36,37]. The central nervous system is involved in the catabolic response of vitamin D, and 1,25-D crosses the blood–brain barrier (BBB) by binding to specific receptors in the brain [38,39].

1,25-Dihydroxycholecalciferol (calcitriol) stimulates calcium and phosphorus absorption by enterocytes after a second hydroxylation step. A similar stimulation process happens in a mechanism with parathormone. Calcitriol interacts with parathormone to influence calcium absorption by osteoclasts [30,40]. According to Landel, Stephan, Cui, et al. endothelial cells and neurons can lead the reaction of converting inactive cholecalciferol to 25(OH)D3: the compound is metabolized by microglia or neurons to 1,25(OH)2D3, which is then translocated to astrocytes, where it can then bind to the VDR and carry out gene transcription. If 1,25(OH)2D3 is produced in excess, it remains inactivated [41].

Studies correlating abnormal human brain development with low vitamin D concentrations in the body are still lacking. In vivo studies in pregnant rats have documented hypovitaminosis D resulting in developmental abnormalities and changes in brain morphology in young rats. Reduced cerebral cortex thickness, enlarged lateral ventricles, and increased brain mass and length were demonstrated in born rats [42]. Hypovitaminosis D modifies neuron length and affects the length and growth process, neurite branching and periaqueductal length [43]. It is believed that a critical window occurs during late pregnancy, where vitamin D deficiency caused a change in the behavioral phenotype of the adult [44].

Research on vitamin D in the human brain concerns the assessment of maternal serum 25(OH)D concentrations and psychological and neurocognitive testing in born offspring. Much of the findings are conflicting, and further studies are needed. In general, children whose mothers had reduced serum vitamin D concentrations during pregnancy demonstrate an increased risk of language disorders and speech problems in later life [45]. Low vitamin D concentrations may also be responsible for the development of schizophrenia and depression [46,47,48,49]. In an older population, changes in the human brain with vitamin D deficiency include increased volume of white matter abnormalities of the brain [50]. Vitamin D plays an indispensable role in the aging process. It influences the maintenance of a proficient level of cognitive functions and acts on neurodegenerative mechanisms in the brain. It is also involved in brain plasticity, neurotransmission, neuromodulation, neuroprotection and vascular risk factors for stroke, among others [13,51,52]. Its deficiency reduces the volume of brain and hippocampus tissue. Vitamin D is involved in a range of neuromodulation mechanisms in the adult brain and hippocampus [13,53,54,55,56,57], as well as in the thalamic nuclei, cerebellar granule cells, medial hypothalamic nucleus, lateral geniculate nucleus, substantia nigra, entorhinal cortex, supraoptic and paraventricular nuclei of the hypothalamus, amygdala or the previously mentioned pyramidal neurons of the hippocampal region [42].

## 4. Vitamin D and Stroke Incidence

Stroke is recognized as the second cause of mortality among human populations worldwide [58]. High serum vitamin D levels have long been associated with improved cardiovascular health. This is of particular importance in the context of reducing the risk of stroke, which is why many authors have proposed that vitamin D level may be a modifiable risk factor [57,59,60]. VDR (vitamin D receptor) has been found in endothelia, vascular smooth muscle, and cardiomyocytes [5,61,62]. Vitamin D affects a large number of genes, including those responsible for cell cycle regulation, proliferation, apoptosis, and angiogenesis. It reduces the progression of atherosclerotic lesions, inhibits the renin–angiotensin system, and may minimize parathormone levels and inflammation. This means that it has a positive effect on the atherosclerotic process [63,64,65,66,67,68,69].

Scientists are increasingly mentioning the link between low vitamin D levels and the incidence of strokes. A meta-analysis by Zhang, Li, Gao showed that higher serum concentrations of 25(OH)D had a protective effect on the total number of cardiovascular events and mortality due to cardiovascular diseases, including stroke [70]. A large population study in China found that low vitamin D levels may be associated with the risk of an ischemic stroke [71]. This is also confirmed by a meta-analysis by Zhou, Wang, Huang et al., which also found that low vitamin D levels were not associated with hemorrhagic stroke [72]. There is no lack of systematic reviews and meta-analyses that deny the protective effect of vitamin D [73] and confirm that low vitamin D levels are associated with an increased risk of stroke, but additional supplements have no impact on risk reduction [74].

**Figure 1:**
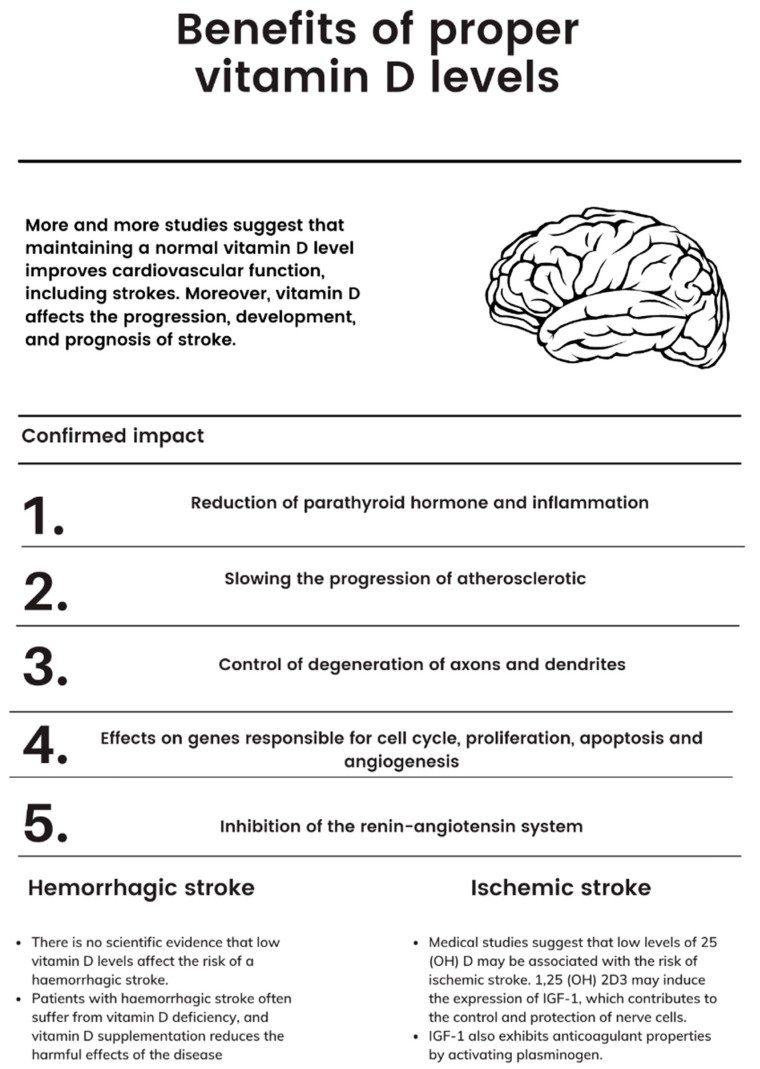
Benefits of proper vitamin D supplementation.

Hemorrhagic stroke patients often suffer from vitamin D deficiency, and vitamin D supplementation reduces the harmful effects of the disease (Figure 1) [7,75,76,77]; however, as these studies use varied methodologies and have small control groups, the results should be interpreted with caution. Although hypervitaminosis D does not occur with sun exposure or vitamin D-rich foods, it can occur following excessive intake of supplements and medications over an extended period of time. Its overdose can lead to hypercalcemia, a buildup of calcium in the blood. This manifests as nausea and vomiting, muscle weakness, and frequent urination, which can lead to kidney problems and heart rhythm disorders [78].

Available studies conclude that low levels of vitamin D influence the development and severity of complications and prognosis in stroke [79,80,81,82]. Its deficiency can increase the stiffness and narrowing of the vessel lumen. This leads to reduced blood flow and an increased risk of obstruction. This is due to activation of the renin–angiotensin–aldosterone system [83]. In the case of ischemic stroke, MRI showed the development of small vessel disease and white matter damage [84]. When supplemented properly, blood pressure is lowered, vasodilation occurs, and blood flow is restored to neurons where the focus of the stroke is located by increasing nitric oxide synthase activity [54,85]. This is particularly seen in hemorrhagic stroke. The anti-inflammatory effect of 1,25(OH)2D3 attenuates cerebral vasospasm as measured by vessel diameter and basilar artery endothelial function [86].

The mechanism of vitamin D in the transition of ischemic stroke has not yet been thoroughly investigated. However, some studies suggest several neuroprotective mechanisms [24,87,88]. 1,25(OH)2D3 can induce the expression of insulin-like growth factor 1 (IGF-1), affecting the control of axon and dendrite degeneration [75,87]. IGF-1 also exerts antithrombotic properties through plasminogen activation [87,89]. Ischemic stroke triggers an acute phase mechanism in the vascular system, causing an increase in inflammatory markers [90]. Widespread tissue damage in brain vessels triggers an inflammatory response resulting in upregulation of fibrinogen in the liver and blood clotting. High fibrinogen levels in patients diagnosed with acute stroke are a sign of brain damage [91]. High serum fibrinogen levels appear to be a risk factor for vitamin D deficiency in acute stroke patients [79]. Moreover, high fibrinogen levels may be a risk factor for low serum vitamin D levels (<50 nmol/L) in patients with ischemic stroke. High levels of fibrinogen increase plasma viscosity, which can lead to low blood flow in the brain and impaired blood perfusion. Ultimately, this can lead to deterioration and worsening of stroke [92,93].

The blood–brain barrier protects nerve tissue from toxic substances or pathogens. It defines the boundaries between the brain and the circulatory system and regulates the exchange of substances from circulation to the brain. Disturbance of the BBB border results in the development of diseases of the central nervous system [94]. Abnormal BBB function facilitates the formation of brain edema and the development of cerebral hemorrhage, which is associated with an increase in the secretion of various cytokines and chemokines. This leads to secondary ischemic injury [24]. Increased production of reactive oxygen species (ROS) during stroke disrupts substance transport: the BBB becomes non-integral and permeable to many substances that were previously properly blocked due to direct damage [24,95]. However, many studies indicate that normal vitamin D concentrations initiate neuroprotective mechanisms that protect against BBB dysfunction caused by an expanding stroke focus [96,97], and more recently, vitamin D exerts protective effects against blood–brain barrier disruption in ischemic stroke in endothelial cells [23,24].

The available literature highlights the important role played by an adequate diet after stroke, which should include vitamin D, folic acid, mecobalamin, and calcium. Proper nutrition during the disease is a decisive factor for the effectiveness of neurological rehabilitation. Diet is a modifiable element of lifestyle, affecting the reduction or increase in the risk of many diseases, including stroke. Post-stroke patients often experience concomitant diseases associated with poor nutrition, such as anemia, diabetes, osteoporosis, and sarcopenia. This demonstrates the urgent need for customized diets for patients during rehabilitation programs [98,99,100].

Research on the effectiveness of vitamin D and prophylactic supplementation in stroke are still limited, and few studies indicate a positive impact on health after ischemic and hemorrhagic strokes. Many studies have small study samples that would need to be expanded and scaled up in the future. Further limitations are diagnostic precision, including the amount of vitamin D administered, the method of administration, and the timing of serum vitamin D testing with regard to geographic location and sun exposure in specific areas of the world.

## 5. Gut–Brain Axis

The gut–brain axis is defined as the system of two-way signaling between the gastrointestinal tract and the nervous system, in which biochemical mediators produced by the intestinal microflora play a key role [101]. The microbiome is believed to modify CNS function by influencing the activity of immune cells, generating neurotransmitters and synthesizing metabolites. It has been postulated that intestinal microflora dysfunction is related to CNS disease, including stroke [102]. Dysbiosis of the intestinal microflora, i.e., changes in its function and composition, correlate with serious risk factors for stroke, such as obesity, metabolic diseases and atherosclerosis [103]. Moreover, it has been shown that disturbances of the intestinal microflora, as the central immune regulator, are associated with a worse prognosis after stroke [104]. Bacterial components (lipopolysaccharides), microflora metabolites such as short-chain fatty acids and trimethylamine *N*-oxide, and inflammatory cells (T cells) are involved in the gut–brain interaction affecting neuroinflammation and post-stroke outcomes [105].

The effect of vitamin D on the microbiota has been extensively studied. Singh et al. assessed the effect of vitamin D supplementation (50,000 IU/week for 12 weeks) on intestinal microbes in 80 healthy women deficient in vitamin D. It was shown that the administration of vitamin D enhanced the diversity of the intestinal microflora, mainly reducing the ratio of Firmicutes to Bacteroidetes (increase in the number of Bacteroidetes with a simultaneous decrease in the number of Firmicutes) [106]. The effect of vitamin D on the intestinal microflora was also confirmed in a pilot clinical study by Charoenngam et al. Similar to Singh, they investigated the gut microbiome following an intervention with oral vitamin D (600, 4000 and 10,000 IU/day) in adults deficient in vitamin D. They observed a dose-dependent reduction in inflammatory bowel disease associated with an increase in commensal bacteria while reducing the amount of pathogens [107]. Altering the diversity of the gut microflora with vitamin D supplementation has also been studied in many diseases, including cystic fibrosis [108], multiple sclerosis [109], autism/autism spectrum disorder [110,111], diabetes, and obesity [112]. Kanhere et al. assessed the effect of a 12-week intervention with high doses of vitamin D (50,000 IU/week) on the composition of the intestinal and respiratory microflora in patients with cystic fibrosis with vitamin D deficiency. It was shown that in the study group, the ratio of commensal to potentially pathogenic bacteria was significantly higher than in the placebo group, which was associated with a better inflammatory response [109]. Similar findings were presented by Cantarel et al., who assessed vitamin D supplementation (5000 IU/day for 90 days) on the intestinal microflora in multiple sclerosis. They also noted a shift in the composition of the intestinal microflora in favor of commensal bacteria [110]. Finally, Hussein et al. investigated the effect of vitamin D (500 IU/kg/day) on the colon cannabinoid 1 receptor (CB1R), which directly connects the gut microflora to metabolic and cognitive disorders, compared to metformin (200 mg/kg/day) in rats. Improvement in cognitive function and a reduction in LPS, PGN and TNF-α levels was demonstrated, suggesting amelioration of intestinal dysbiosis and neuroinflammation in rats administered vitamin D [112].

The mechanism of action of vitamin D on the microbiome is not fully understood; however, it has been postulated that vitamin D and the intestinal microflora exert a mutual influence. It may be related to the activation of antimicrobial peptide (AMP) expression and support of the epithelial barrier in the intestinal mucosa, or possibly to the enhancement of VDR expression and serum levels of vitamin B by intestinal bacteria [113]. AMP synthesis occurs through the TLR1/2 pathway, which depends, inter alia, on the level of VDR expression [114]. Thus, the activation of AMP generation promotes the inhibition of intestinal dysbiosis, leading to an increased formation of the commensal microbiome [115]. In addition, vitamin D has been shown to modulate intestinal epithelial homeostasis by increasing the expression of proteins involved in maintaining its integrity: occludin, claudin, vinulin and zonula occludens [116].

The available data suggest that vitamin D has an influence on the intestinal microflora, which in turn is related to the prognosis and post-stroke outcomes. Therefore, it seems justified to undertake a well-planned clinical trial that could confirm both the validity of the use of vitamin D in post-stroke patients and explain the mechanism of its action in this group of patients.

## 6. The Effect of Vitamin D Supplementation on Post-Stroke Rehabilitation

Vitamin D deficiency may be associated with an increased risk of stroke onset, severity, and future prognosis. It also affects cognitive decline and physical performance, which is seen in stroke patients who have poorer outcomes in neurological rehabilitation [117]. Because of the many limitations of serum vitamin D testing and the method and amount of its administration, there is little certainty that it will improve rehabilitation outcomes in stroke survivors in every case [28]. Many studies are optimistic and show results in which vitamin D supplementation after stroke improved quality of life levels and facilitated return to normal life in patients (Table 1 and Table 2). A small sample study shows improved outcomes for patients supplementing with vitamin D after stroke [118,119].

Two small, open-label studies conducted by Gupta, Prabhakar, Modi et al. in India have shown that intramuscular injection of high-dose cholecalciferol (600,000 IU) improves scores on various stroke scales and increases survival of post-stroke patients. A randomized, controlled, open-label study included 73 patients with acute ischemic stroke. Each patient was tested for serum (25(OH)D) levels before entering the study. A total of 53 patients with baseline 25(OH)D < 75 nmol/L were randomly assigned to two trials. The first group received an intramuscular injection of 600,000 IU of cholecalciferol once and oral cholecalciferol at a dose of 60,000 IU once per month with one gram of elemental calcium daily along with usual post-stroke care. The second control group received only usual hospital care. Serum vitamin D and iPTH levels were tested at 3 and 6 months of the study, and the follow-up itself was 6 months. The modified Rankin Scale (mRS) was used in the study, and high scores were obtained after 6 months. This confirms the beneficial effect of vitamin D supplementation in post-stroke patients [117].

A randomized, controlled, unblinded study by Narasimhan and Balasubramanian compared outcomes in ischemic stroke patients taking vitamin D supplementation with patients without supplementation. The study used the Scandinavian Stroke Scale (SSS), a reliable and widely used scale in patients with ischemic cerebrovascular disease. The first group of patients received a dose of 6 lac UI of cholecalciferol administered by intramuscular injection; the second group did not receive vitamin D. Patients in both groups were examined at the beginning of the study and after three months. The findings indicate a significant improvement in the outcome among post-stroke patients supplementing with vitamin D [119]. Vitamin D supplementation in patients post-stroke had a beneficial effect in improving rehabilitation outcomes.

A small Japanese study by Momosaki, Abo, Urashima et al. with 100 patients showed no improvement in stroke patients treated with vitamin D. After randomization, each of the 100 subjects took an oral form of vitamin D3 at a dose of 2000 IU/day or a placebo. Each patient received 450 capsules, with each individual capsule containing 400 IU of vitamin D3, meaning that patients took the capsules five times a day. Vitamin D3 was always taken at the same time, after lunch. The study containing rehabilitation and vitamin D supplementation lasted 8 weeks. During patient admission and 8 weeks later at discharge, rehabilitation staff assessed each patient’s Barthel index, Brunnstrom grade (arm, hand, and leg on the affected side), handgrip strength (bilaterally), and calf circumference (bilaterally). A total of 97 patients completed the Japanese study.

There was improvement in Barthel Index scores at week 8 of rehabilitation as the primary endpoint; secondary outcomes were observed in Barthel Index performance, hand compression strength, and calf circumference. No differences in other secondary endpoints were found between groups. None of these differences were statistically significant, indicating that daily supplementation with 2000 IU of vitamin D3 in patients after acute stroke was ineffective and did not provide the intended benefits. One potential error was that serum 25-hydroxyvitamin D levels were not considered as an inclusion criterion when qualifying patients for the study; the researchers justify this with the assumption that almost all elderly patients undergoing rehabilitation have vitamin D deficiency, and therefore, the deficiency study itself was not included in the patients participating in the study [122,123]. Other limitations also highlighted by the authors include the small study group, the short duration of supplementation and the entire study, making it impossible to accurately determine the long-term and sporadic role of vitamin D supplementation in stroke patients. Another extremely important limitation was the comparison of only one vitamin D sample with a placebo sample [28].

The 2021 study by Torrisi, Bonanno, Formica et al. improvements have been observed in stroke patients undergoing intensive neurorehabilitation and vitamin D supplementation, as well as after neurorehabilitation itself. A randomized, double-blind, parallel, monocentric study lasting 12 weeks was performed involving 40 patients after ischemic and hemorrhagic strokes. Participants were assigned randomly, in a 1:1 proportion, between two parallel groups: the experimental group in which 2000 IU/day of cholecalciferol was administered orally, and the control group in which patients received no vitamin D supplementation. All patients enrolled in the study performed intensive neurorehabilitation consisting of cognitive and motor training. All patients completed the training. All study participants were screened in two stages, at the beginning and at the end of rehabilitation. Patients were assessed using the GSE scale, the Montgomery Aasberg Depression Rating Scale (MADRS), and the Functional Independence Measure (FIM). Serum vitamin D and calcium levels were monitored. Significant improvements were observed in patients in the experimental group as well as the control group in both psychological and functional performance. The patients taking vitamin D supplementation showed greater variability than patients who did not. The results indicate that intensive neurorehabilitation has a beneficial effect on functional recovery after stroke; in addition, a clear improvement was shown in the experimental group, suggesting that vitamin D supplementation may also play a role [120]. However, vitamin D supplementation in stroke patients did not significantly improve patient outcomes.

According to Karasu and Karataş, vitamin D supplementation can increase the effectiveness of rehabilitation in post-stroke patients. This is especially important in patients who are in the first three months after stroke and who will receive neurological rehabilitation for the first time. The retrospective study included 76 stroke patients. Patients in the study experienced a stroke (ischemic/hemorrhagic) for the first time in their lives. The Brunnstrom Recovery Stage (BRS) for the lower limb and the Functional Assessment of Movement (FAC) scale were used to measure outcomes in terms of motor function. Serum 25-hydroxyvitamin D (25(OH)D) levels measured in ng/mg were examined during the first week of the study. Patients were divided into two groups: those undergoing vitamin D supplementation during rehabilitation as well as those who did not receive this supplementation. For 4–12 weeks, patients took oral vitamin D (50,000 IU) while receiving rehabilitation, and the total vitamin D dose ranged from 200,000 to 600,000 IU. Vitamin D levels before rehabilitation and BRS and FAC scores, as well as changes in scores before and after the rehabilitation process, in stroke patients were recorded and compared in both the control and study groups. After the rehabilitation process, there was a positive and statistically significant change in FAC and BRS scores in the group receiving vitamin D. In addition, we compared the effect of vitamin D supplementation on FAC and BRS scores in patients who started rehabilitation treatment within the first 3 months after stroke. It was found that the change in FAC and BRS scores was statistically significant in patients treated with vitamin D. This demonstrates the beneficial effect on vitamin D intake in patients during the rehabilitation process after stroke. Vitamin D supplementation during post-stroke rehabilitation may have a positive effect on lower limb mobility and motor function according to Karasu and Karataş [121]. In this study, vitamin D supplementation in stroke patients had a positive effect, and patients had better rehabilitation outcomes.

Sari, Durmus, Karaman et al. investigated the effects of vitamin D supplementation on rehabilitation outcomes and balance in patients with hemiplegia due to ischemic stroke was examined. Seventy-two ischemic stroke patients with low blood levels of vitamin D hospitalized for hemiplegia rehabilitation were included in the study. A division was made into two groups. Group A received vitamin D by intramuscular injection (300,000 IU vitamin D). Group B received saline by intramuscular injection. Patients were examined at the beginning of the study process and at the third month. Brunnstrom scale, modified Barthel index, Berg balance scale and functional ambulation scale (FAS) were used to examine the effects. By the end of the third month, a significant difference was found between the two groups in the modified Barthel index and Berg balance scale. No statistically significant change was observed in Brunnstrom scale or functional ambulation scale (FAS) scores. In patients after ischemic stroke, vitamin D supplementation (300,000 IU) did not significantly affect motor recovery and mobility. The study confirms that vitamin D supplementation accelerated recovery and increased activity levels in patients. This confirms the validity that it would be appropriate to extend follow-up studies with more patients after stroke [118]. Vitamin D supplementation in post-stroke patients did not clearly improve patient outcomes.

## 7. Discussion

There is a lack of consistency in the results achieved between studies investigating the combination of vitamin D supplementation in stroke patients and improving rehabilitation outcomes. Research has many limitations. Sometimes, serum 25-hydroxyvitamin D levels are not measured in patients qualified for the study, which means that the authors may qualify patients with high or normal vitamin D levels rather than deficiency [28]. Another major limitation is sample sizes, which are often too small. A larger number of participants could have altered the outcome, and the authors examine different models, regimens of how to administer, amount of vitamin D, or even longer treatment times for patients. The short study period was not able to examine the long-term effects of supplementation [28,118,119,120,121,122]. There is also a lack of expanded research on vitamin D supplementation by type of stroke (ischemic/hemorrhagic) in patients. Zhou, Wang, Huang et al. published a systematic review and meta-analysis of studies examining the possible association of vitamin D with stroke risk [72]. They confirmed the hypothesis that lower vitamin D levels are associated with an increased risk of ischemic stroke but not hemorrhagic stroke. This shows how important it is to increase research and diversity on this phenomenon. A retrospective review conducted by Bakradze, McCullough, Staff et al. indicated a direct correlation between elderly and undernourished patients. Patients were at increased risk of severe clinical ICH (intracerebral hemorrhage) [124]. There is also a lack of broad studies that consider ethnic and racial determinants. A study by Judd, Morgan, Panwar et al. indicated that lower 25(OH)D levels are an important risk factor for stroke in adults, regardless of black or white race [97]. In contrast, a study by Michos, Reis, Post showed that in white race people, vitamin D deficiency was associated with an increased risk of stroke death. No similar relationship was found in black race people, although a higher stroke mortality rate was detected, but it was not associated with low vitamin D levels and stroke incidence [60]. Studies showing genetic predisposition for absorption and metabolism of vitamin D are inconclusive. The study by Türkanoğlu Özçelik, Öner, Can Demirdöğen et al. examined the genetic polymorphisms *CYP24A1 rs927 650* and *CYP2R1 rs10 741 657*. Polymorphic genotypes of polymorphisms together with hypertension, diabetes, smoking and obesity were considered relevant to the risk of ischemic stroke [125]. There is a need for further studies taking into account the presence or absence of *CYP2R1*, *CYP27B1*, *CYP24A1* and VDR polymorphisms [31].

## 8. Conclusions

There is growing evidence that vitamin D has a positive impact on the prevention of cardiovascular disease and contributes to better rehabilitation outcomes in stroke patients. Numerous studies testing the efficacy of vitamin D supplementation in post-stroke patients are faced with many limitations affecting the results. Due to the low number of studies and other limitations, it is not unambiguous that vitamin D supplementation in stroke patients always has a positive effect on improving rehabilitation. Considering that stroke is the first cause of disability and that older adults have high vitamin D deficiencies in their bodies, it is worth expanding the studies testing the effectiveness of vitamin D supplementation. Future studies should be controlled and randomized with a large sample of more than 1000 patients with at least 5 years of follow-up and vitamin D supplementation.

## Figures and Tables

**Table 1:** Criteria for study entry for different authors.

Study, Year, Reference	Types of Criteria					
	Type of Stroke	Testing Serum Vitamin D Levels before the Study	Age	How to Evaluate Vitamin D	Exclusions	Other
**Narasimhan et al., 2017 [119].**	Ischemic, middle cerebral artery ischemia	Serum 25-hydroxy vitamin D level 21–29 ng/mL and Serum 25-hydroxy vitamin D level ≤ 20 ng/mL	50–80 years	Measurement of serum 25-hydroxyvitamin D by electrochemiluminescence binding assay. Interpretation of the result according to the US Endocrine Society	Hemorrhagic stroke, large MCA stroke, lacunar stroke, thrombolytic therapy, very poor general condition, multiple organ failure, normal vitamin D levels (serum 25-hydroxyvitamin D concentration ≥ 30 ng/mL)	Diagnosis of stroke, clinical evaluative, CT, MRI
**Momosaki et al., 2019 [28].**	Cerebral infarction, intracerebral hemorrhage or subarachnoid hemorrhage	Serum 25-hydroxyvitamin D level was not included as an inclusion criterion	At least 20 years	Irrelevant	History of stones in the urinary tract, vitamin D3 or activated vitamin D supplementation before stroke, osteoporosis, bone structure, dysphagia, or other disorder that would make it difficult to take an oral vitamin D supplement, inability to participate in the study in the opinion of the attending physiologist	First stroke in life, admission to the convalescent rehabilitation unit after acute stroke treatment, deemed by the attending physiologist to require 8 weeks of inpatient rehabilitation
**Torrisi et al., 2021 [120].**	Ischemic/hemorrhage stroke	Irrelevant, Serum 25-hydroxyvitamin D levels, were tested, but this was not a criterion	Irrelevant	Irrelevant	Mini Mental State Examination < 15, psychiatric conditions/treatment with antidepressants, patients already on vitamin D supplementation, also in combination with calcium, multivitamins or other medications, and conditions that do not allow for a neurorehabilitation program	Stroke that occurred between 30 and 60 days before, eligibility for rehabilitation treatment
**Karasu et al., 2021 [121].**	Ischemic/hemorrhage stroke	Serum 25-hydroxyvitamin D (25(OH)D) levels measured in ng/mL	Irrelevant	Irrelevant	No pre-rehabilitation measurement of vitamin D levels, chronic kidney, liver, or lung disease that may affect vitamin D levels, current steroid treatment, previous orthopedic problems known to affect lower extremity function	First stroke in life, diagnosis of stroke, clinical evaluative, CT, MRI, inpatient stroke rehabilitation treatment May 2018–February 2020
**Sari et al., 2018 [118].**	Ischemic stroke	Measured during winter, <30 ng/mL	Irrelevant	Measurement of 25 (OH) vitamin D3 using the RIA CT kit (BioSource Europe SA, Nivelles, Belgium) by radioimmunoassay	End-stage disease (cancer) or disease other than stroke that may affect balance or mobility (e.g., multiple sclerosis, Parkinson’s disease, or pelvic and lower extremity surgery), limit sun exposure (e.g., acquired vitiligo and psoriasis), or affect vitamin D levels (e.g., chronic renal failure and celiac disease)	Current hemiplegia after stroke, Hospitalization for neurological rehabilitation (hemiplegia) between September 2014 and March 2015, no cerebrovascular disease, at least a 2-month gap that has passed since the last stroke
**Gupta et al., 2016 [117].**	Ischemic stroke	25(OH)D concentration < 75 nmol/L	Age ≥ 35 years	Serum 25(OH)D concentration measured by chemiluminescence	Previously taken vitamin D and calcium supplementation, thrombolysis performed, kidney and liver dysfunction	MRS (Modified Rankin Score) before stroke < 2, diagnosis of stroke, CT, MRI,

**Table 2:** Comparison of studies on vitamin D supplementation in patients after stroke.

Study, Year, Reference	Type of Study	Duration of the Study	Amount and Method of Administration of Vitamin D	Scales, Tests	Results	Conclusions
**Narasimhan et al., 2017 [119].**	Randomized, controlled, unblinded	3 months	Single dose of 6 lac IU cholecalciferol, intramuscular injection IIM)	Scandinavian stroke scale (SSS)—stroke severity assessment	The differences in SSS from admission and after three months in group A—study (6.39 ± 4.56) and group B—control (2.50 ± 2.20) were statistically analyzed and found to be highly significant (*p* < 0.001)	After three months, there was a significant improvement in stroke outcome in patients who received vitamin D
**Momosaki et al., 2019 [28]**.	Randomized, multicenter, double-blind, placebo-controlled trial	8 weeks	Vitamin D3, 400 IU, 5 times daily (2000 IU vit. D3 per day), oral tablets	Barthel Index score, Brunnstrom stage (arm, hand, and leg on the affected side), hand grip strength (bilaterally), and calf circumference (bilaterally)	The mean (±standard deviation) increase in Barthel Index score was 19.0 ± 14.8 in the supplementation group and 19.5 ± 13.1 in the placebo group (*p* = 0.88). The effectiveness of the Barthel Index was 0.32 ± 0.31 in the supplementation group and 0.28 ± 0.21 in the placebo group (*p* = 0.38). There were no differences between groups in other secondary endpoints	Oral vitamin D3 supplementation does not improve rehabilitation outcomes after acute stroke.
**Torrisi et al., 2021 [120].**	Randomized, double-blind, parallel, monocentric, clinical	3 months	2000 IU/day cholecalciferol, oral	Montgomery Aasberg Depression Rating Scale (MADR), Functional Independent Measures (FIM)	In the vitamin D group, we highlighted significant differences between T0 and T1 in calcium (*p* < 0.001), vitamin D (*p* < 0.001), MADR (*p* = 0.001), and FIM (*p* < 0.001). In the health control group, we found a significant difference in calcium (*p* = 0.003), vitamin D (*p* < 0.001), MADR (*p* = 0.006), overall sense of self-efficacy (*p* = 0.009), and in FIM (*p* < 0.001)	Beneficial effects on improved mood and function are mainly due to neurorehabilitation rather than vitamin D supplementation
**Karasu et al., 2021 [121].**	Retrospective	3 months	Weekly vitamin D supplementation (50,000 IU) for 4–12 weeks, orally, total vitamin D intake ranged from 200,000 to 600,000 IU	Brunnstrom recovery stage (lower extremity), (BRS), functional ambulation classification (FAC)	At the end of rehabilitation, the change in FAC and Brunnstrom scores was higher in patients receiving vitamin D supplementation (*p* = 0.005 *p* = 0.018). The change in FAC and Brunnstrom scores in patients undergoing rehabilitation for the first time and/or within the first 3 months after stroke was higher in the group receiving vitamin D supplementation compared with the group not receiving vitamin D (*p* < 0.05)	Vitamin D supplementation may increase the effectiveness of rehabilitation therapy in patients during the first 3 months after stroke
**Sari et al., 2018 [118].**	Randomized, double-blind, placebo-controlled	3 months	300,000 IU Vitamin D, 2 mL fluid, intramuscular injection (IM)	Brunnstrom recovery staging (BRS), functional ambulation scale (FAS), modified Barthel index (MBI) scores, Berg balance scale (BBS)	By the end of the third month, The Berg balance scale results and modified Barthel index scores significantly differed between the two groups, whereas Brunnstrom recovery staging and functional ambulation scale test results did not.	Vitamin D administration increased activity levels and accelerated recovery, but did not significantly affect movement or motor recovery
**Gupta et al., 2016 [117].**	Randomized, controlled, open-label	6 months	Single intramuscular injection of 600,000 IU cholecalciferol, oral cholecalciferol 60,000 IU once a month with one gram elemental calcium daily	Modified Rankin scale (mRS)	Serum 25(OH)D levels increased by 47.3 (25.0–69.5) nmol/L in the vitamin D and calcium supplementation group (*p* < 0.001) and by 0.8 (−7.5–8.8) nmol/L in the group receiving usual care without supplementation (*p* = 0.86) up to 6 months. Patients who received usual care were 2–3 times more likely to be vitamin D deficient/deficient at 6 months compared with those who received vitamin D and calcium supplementation	After 6 months 11 patients (52.4%)—mRS score between 0 and 2 in the vitamin D plus calcium-supplemented arm and 10 patients (43.5%) had a good outcome in the usual care arm, Adjusted OR 1.9, 95% CI 0.6–6.4; *p* = 0.31, Risk difference 4.7%

## References

[1] Deluca H.F. (2014). History of the discovery of vitamin D and its active metabolites. BoneKEy Rep..

[2] Muscogiuri G., Barrea L., Scannapieco M., Di Somma C., Scacchi M., Aimaretti G., Savastano S., Colao A., Marzullo P. (2019). The lullaby of the sun: The role of vitamin D in sleep disturbance. Sleep Med..

[3] Suvarna V., Mohan V. (2020). Vitamin D and its role in coronavirus disease 2019 (COVID-19). J. Diabetol..

[4] Rafeeq H., Ahmad S., Tareen M.B.K., Shahzad K., Bashir A., Jabeen R., Tariq S., Shehzadi I. (2020). Biochemistry of Fat Soluble Vitamins, Sources, Biochemical Functions and Toxicity. J. Life Sci..

[5] Holick M.F. (2007). Vitamin D deficiency. N. Engl. J. Med..

[6] Melamed M.L., Michos E.D., Post W., Astor B. (2008). 25-hydroxyvitamin D levels and the risk of mortality in the general population. Arch. Intern. Med..

[7] Ashouri R., Fangman M., Brielmaier J., Fields Z.A., Campo N., Doré S. (2021). Nutritional Supplementation of Naturally Occurring Vitamin D to Improve Hemorrhagic Stroke Outcomes. Front. Neurol..

[8] Holick M.F. (2005). Vitamin D: Important for prevention of osteoporosis, cardiovascular heart disease, type 1 diabetes, autoimmune diseases, and some cancers. South. Med. J..

[9] Brøndum-Jacobsen P., Benn M., Jensen G.B., Nordestgaard B.G. (2012). 25-hydroxyvitamin d levels and risk of ischemic heart disease, myocardial infarction, and early death: Population-based study and meta-analyses of 18 and 17 studies. Arterioscler. Thromb. Vasc. Biol..

[10] Moan J., Dahlback A., Ma L., Juzeniene A. (2009). Influenza, solar radiation and vitamin D. Derm.-Endocrinol..

[11] Laaksi I., Ruohola J.P., Mattila V., Auvinen A., Ylikomi T., Pihlajamäki H. (2010). Vitamin D supplementation for the prevention of acute respiratory tract infection: A randomized, double-blinded trial among young Finnish men. J. Infect. Dis..

[12] Tripkovic L., Lambert H., Hart K., Smith C.P., Bucca G., Penson S., Chope G., Hyppönen E., Berry J., Vieth R. (2012). Comparison of vitamin D2 and vitamin D3 supplementation in raising serum 25-hydroxyvitamin D status: A systematic review and meta-analysis. Am. J. Clin. Nutr..

[13] Aspell N., Lawlor B., O’Sullivan M. (2018). Is there a role for vitamin D in supporting cognitive function as we age?. Proc. Nutr. Soc..

[14] Holick M.F. (2004). Sunlight and vitamin D for bone health and prevention of autoimmune diseases, cancers, and cardiovascular disease. Am. J. Clin. Nutr..

[15] Fraikin G.Y. (2018). Signaling Mechanisms Regulating Diverse Plant Cell Responses to UVB Radiation. Biochemistry.

[16] Nair R., Maseeh A. (2012). Vitamin D: The “sunshine” vitamin. J. Pharmacol. Pharmacother..

[17] Schuster I. (2011). Cytochromes P450 are essential players in the vitamin D signaling system. Biochim. Biophys. Acta (BBA)-Proteins Proteom..

[18] Ross A.C., Taylor C.L., Yaktine A.L., Del Valle H.B., Institute of Medicine (US) Committee to Review Dietary Reference Intakes for Vitamin D and Calcium (2011). The national academies collection: Reports funded by national institutes of health. Dietary Reference Intakes for Calcium and Vitamin D.

[19] Holick M.F. (2008). Vitamin D: A D-Lightful health perspective. Nutr. Rev..

[20] Holick M.F. (2006). Resurrection of vitamin D deficiency and rickets. J. Clin. Investig..

[21] Holick M.F. (2010). The Vitamin D Deficiency Pandemic: A Forgotten Hormone Important for Health. Public Health Rev..

[22] Siniscalchi A., Lochner P., Anticoli S., Chirchiglia D., De Sarro G., Gallelli L. (2019). What is the Current Role for Vitamin D and the Risk of Stroke?. Curr. Neurovasc. Res..

[23] Yarlagadda K., Ma N., Doré S. (2020). Vitamin D and Stroke: Effects on Incidence, Severity, and Outcome and the Potential Benefits of Supplementation. Front. Neurol..

[24] Won S., Sayeed I., Peterson B.L., Wali B., Kahn J.S., Stein D.G. (2015). Vitamin D prevents hypoxia/reoxygenation-induced blood-brain barrier disruption via vitamin D receptor-mediated NF-kB signaling pathways. PLoS ONE.

[25] Afzal S., Nordestgaard B.G. (2017). Vitamin D, Hypertension, and Ischemic Stroke in 116 655 Individuals from the General Population: A Genetic Study. Hypertension.

[26] Sheerah H.A., Eshak E.S., Cui R., Imano H., Iso H., Tamakoshi A. (2018). Relationship Between Dietary Vitamin D and Deaths From Stroke and Coronary Heart Disease: The Japan Collaborative Cohort Study. Stroke.

[27] Gepner A.D., Ramamurthy R., Krueger D.C., Korcarz C.E., Binkley N., Stein J.H. (2012). A prospective randomized controlled trial of the effects of vitamin D supplementation on cardiovascular disease risk. PLoS ONE.

[28] Momosaki R., Abo M., Urashima M. (2019). Vitamin D Supplementation and Post-Stroke Rehabilitation: A Randomized, Dou-ble-Blind, Placebo-Controlled Trial. Nutrients.

[29] Tague S.E., Smith P.G. (2011). Vitamin D receptor and enzyme expression in dorsal root ganglia of adult female rats: Modulation by ovarian hormones. J. Chem. Neuroanat..

[30] Anderson P.H., May B.K., Morris H.A. (2003). Vitamin D metabolism: New concepts and clinical implications. Clin. Biochem. Rev..

[31] Siotto M., Santoro M., Aprile I. (2020). Vitamin D and Rehabilitation after Stroke: Status of Art. Appl. Sci..

[32] Wikvall K. (2001). Cytochrome P450 enzymes in the bioactivation of vitamin D to its hormonal form (review). Int. J. Mol. Med..

[33] Prosser D.E., Jones G. (2004). Enzymes involved in the activation and inactivation of vitamin D. Trends Biochem. Sci..

[34] Eelen G., Verlinden L., van Camp M., van Hummelen P., Marchal K., de Moor B., Mathieu C., Carmeliet G., Bouillon R., Verstuyf A. (2004). The effects of 1alpha,25-dihydroxyvitamin D3 on the expression of DNA replication genes. J. Bone Miner. Res..

[35] Peterlik M., Cross H.S. (2009). Vitamin D and calcium insufficiency-related chronic diseases: Molecular and cellular pathophysiology. Eur. J. Clin. Nutr..

[36] Eyles D.W., Smith S., Kinobe R., Hewison M., McGrath J.J. (2005). Distribution of the vitamin D receptor and 1 alpha-hydroxylase in human brain. J. Chem. Neuroanat..

[37] Zelzer S., Meinitzer A., Herrmann M., Goessler W., Enko D. (2021). A Novel Method for the Determination of Vitamin D Metabolites Assessed at the Blood-Cerebrospinal Fluid Barrier. Biomolecules.

[38] DeLuca G.C., Kimball S.M., Kolasinski J., Ramagopalan S.V., Ebers G.C. (2013). Review: The role of vitamin D in nervous system health and disease. Neuropathol. Appl. Neurobiol..

[39] Kalueff A.V., Minasyan A., Keisala T., Kuuslahti M., Miettinen S., Tuohimaa P. (2006). The vitamin D neuroendocrine system as a target for novel neurotropic drugs. CNS Neurol. Disord.-Drug Targets.

[40] Bivona G., Agnello L., Ciaccio M. (2018). The immunological implication of the new vitamin D metabolism. Cent. Eur. J. Immunol..

[41] Landel V., Stephan D., Cui X., Eyles D., Feron F. (2018). Differential expression of vitamin D-associated enzymes and receptors in brain cell subtypes. J. Steroid. Biochem. Mol. Biol..

[42] Eyles D., Brown J., Mackay-Sim A., McGrath J., Feron F. (2003). Vitamin D3 and brain development. Neuroscience.

[43] Marini F., Bartoccini E., Cascianelli G., Voccoli V., Baviglia M.G., Magni M.V., Garcia-Gil M., Albi E. (2010). Effect of 1alpha,25-dihydroxyvitamin D3 in embryonic hippocampal cells. Hippocampus.

[44] O’Loan J., Eyles D.W., Kesby J., Ko P., McGrath J.J., Burne T.H. (2007). Vitamin D deficiency during various stages of pregnancy in the rat; its impact on development and behaviour in adult offspring. Psychoneuroendocrinology.

[45] Whitehouse A.J., Holt B.J., Serralha M., Holt P.G., Kusel M.M., Hart P.H. (2012). Maternal serum vitamin D levels during pregnancy and offspring neurocognitive development. Pediatrics.

[46] Lucas R.M., Ponsonby A.L., Pasco J.A., Morley R. (2008). Future health implications of prenatal and early-life vitamin D status. Nutr. Rev..

[47] McGrath J., Welham J., Pemberton M. (1995). Month of birth, hemisphere of birth and schizophrenia. Br. J. Psychiatry.

[48] Maddock J., Berry D.J., Geoffroy M.C., Power C., Hyppönen E. (2013). Vitamin D and common mental disorders in mid-life: Cross-sectional and prospective findings. Clin. Nutr..

[49] Armstrong D.J., Meenagh G.K., Bickle I., Lee A.S., Curran E.S., Finch M.B. (2007). Vitamin D deficiency is associated with anxiety and depression in fibromyalgia. Clin. Rheumatol..

[50] Annweiler C., Bartha R., Karras S.N., Gautier J., Roche F., Beauchet O. (2015). Vitamin D and white matter abnormalities in older adults: A quantitative volumetric analysis of brain MRI. Exp. Gerontol..

[51] van Schoor N.M., Comijs H.C., Llewellyn D.J., Lips P. (2016). Cross-sectional and longitudinal associations between serum 25-hydroxyvitamin D and cognitive functioning. Int. Psychogeriatr..

[52] Wilson V.K., Houston D.K., Kilpatrick L., Lovato J., Yaffe K., Cauley J.A., Harris T.B., Simonsick E.M., Ayonayon H.N., Kritchevsky S.B. (2014). Relationship between 25-hydroxyvitamin D and cognitive function in older adults: The Health, Aging and Body Composition Study. J. Am. Geriatr. Soc..

[53] Croll P.H., Boelens M., Vernooij M.W., van de Rest O., Zillikens M.C., Ikram M.A., Voortman T. (2021). Associations of vitamin D deficiency with MRI markers of brain health in a community sample. Clin. Nutr..

[54] Buell J.S., Dawson-Hughes B. (2008). Vitamin D and neurocognitive dysfunction: Preventing “D”ecline?. Mol. Asp. Med..

[55] Brown J., Bianco J.I., McGrath J.J., Eyles D.W. (2003). 1,25-dihydroxyvitamin D3 induces nerve growth factor, promotes neurite outgrowth and inhibits mitosis in embryonic rat hippocampal neurons. Neurosci. Lett..

[56] Garcion E., Wion-Barbot N., Montero-Menei C.N., Berger F., Wion D. (2002). New clues about vitamin D functions in the nervous system. Trends Endocrinol. Metab..

[57] Wang T.J., Pencina M.J., Booth S.L., Jacques P.F., Ingelsson E., Lanier K., Benjamin E.J., D’Agostino R.B., Wolf M., Vasan R.S. (2008). Vitamin D deficiency and risk of cardiovascular disease. Circulation.

[58] Talebi A., Amirabadizadeh A., Nakhaee S., Ahmadi Z., Mousavi-Mirzaei S.M. (2020). Cerebrovascular disease: How serum phosphorus, vitamin D, and uric acid levels contribute to the ischemic stroke. BMC Neurol..

[59] Perna L., Schöttker B., Holleczek B., Brenner H. (2013). Serum 25-hydroxyvitamin D and incidence of fatal and nonfatal cardio-vascular events: A prospective study with repeated measurements. J. Clin. Endocrinol. Metab..

[60] Michos E.D., Reis J.P., Post W.S., Lutsey P.L., Gottesman R.F., Mosley T.H., Sharrett A.R., Melamed M.L. (2012). 25-Hydroxyvitamin D deficiency is associated with fatal stroke among whites but not blacks: The NHANES-III linked mor-tality files. Nutrition.

[61] Nibbelink K.A., Tishkoff D.X., Hershey S.D., Rahman A., Simpson R.U. (2007). 1,25(OH)2-vitamin D3 actions on cell proliferation, size, gene expression, and receptor localization, in the HL-1 cardiac myocyte. J. Steroid Biochem. Mol. Biol..

[62] Tarcin O., Yavuz D.G., Ozben B., Telli A., Ogunc A.V., Yuksel M., Toprak A., Yazici D., Sancak S., Deyneli O. (2009). Effect of vitamin D deficiency and replacement on endothelial function in asymptomatic subjects. J. Clin. Endocrinol. Metab..

[63] Dusso A.S., Brown A.J., Slatopolsky E. (2005). Vitamin D. Am. J. Physiol. Ren. Physiol..

[64] Nagpal S., Na S., Rathnachalam R. (2005). Noncalcemic actions of vitamin D receptor ligands. Endocr. Rev..

[65] Masuda S., Jones G. (2006). Promise of vitamin D analogues in the treatment of hyperproliferative conditions. Mol. Cancer Ther..

[66] Li Y.C., Kong J., Wei M., Chen Z.F., Liu S.Q., Cao L.P. (2002). 1,25-Dihydroxyvitamin D(3) is a negative endocrine regulator of the renin-angiotensin system. J. Clin. Investig..

[67] Khundmiri S.J., Murray R.D., Lederer E. (2016). PTH and Vitamin D. Compr. Physiol..

[68] Yin K., You Y., Swier V., Tang L., Radwan M.M., Pandya A.N., Agrawal D.K. (2015). Vitamin D Protects Against Atherosclerosis via Regulation of Cholesterol Efflux and Macrophage Polarization in Hypercholesterolemic Swine. Arterioscler. Thromb. Vasc. Biol..

[69] Koyama T., Shibakura M., Ohsawa M., Kamiyama R., Hirosawa S. (1998). Anticoagulant effects of 1alpha,25-dihydroxyvitamin D3 on human myelogenous leukemia cells and monocytes. Blood.

[70] Zhang R., Li B., Gao X., Tian R., Pan Y., Jiang Y., Gu H., Wang Y., Wang Y., Liu G. (2017). Serum 25-hydroxyvitamin D and the risk of cardiovascular disease: Dose-response meta-analysis of prospective studies. J. Clin. Nutr..

[71] Leung R.Y.H., Han Y., Sing C.-W., Cheung B.M., Wong I.C., Tan K.C., Kung A.W.C., Cheung C.L. (2017). Serum 25-hydroxyvitamin D and the risk of stroke in Hong Kong Chinese. Thromb. Haemost..

[72] Zhou R., Wang M., Huang H., Li W., Hu Y., Wu T. (2018). Lower Vitamin D Status Is Associated with an Increased Risk of Ischemic Stroke: A Systematic Review and Meta-Analysis. Nutrients.

[73] Barbarawi M., Kheiri B., Zayed Y., Barbarawi O., Dhillon H., Swaid B., Yelangi A., Sundus S., Bachuwa G., Alkotob M.L. (2019). Vitamin D Supplementation and Cardiovascular Disease Risks in More Than 83 000 Individuals in 21 Randomized Clinical Trials: A Meta-analysis. JAMA Cardiol..

[74] Su C., Jin B., Xia H., Zhao K. (2021). Association between Vitamin D and Risk of Stroke: A PRISMA-Compliant Systematic Review and Meta-Analysis. Eur. Neurol..

[75] Yalbuzdag S.A., Sarifakioglu B., Afsar S.I., Celik C., Can A., Yegin T., Senturk B., Guzelant A.Y. (2015). Is 25(OH)D Associated with Cognitive Impairment and Functional Improvement in Stroke? A Retrospective Clinical Study. J. Stroke Cerebrovasc. Dis..

[76] Kilkkinen A., Knekt P., Aro A., Rissanen H., Marniemi J., Heliövaara M., Impivaara O., Reunanen A. (2009). Vitamin D status and the risk of cardiovascular disease death. Am. J. Epidemiol..

[77] Makariou S.E., Michel P., Tzoufi M.S., Challa A., Milionis H.J. (2014). Vitamin D and stroke: Promise for prevention and better outcome. Curr. Vasc. Pharmacol..

[78] Asif A., Farooq N. (2022). Vitamin D toxicity. StatPearls.

[79] Miao H., Zhu H., Luan X., Huang G., Chen M., Yuan Z., Wang Z. (2020). Risk Factors of Vitamin D Deficiency in Chinese Is-chemic Stroke Patients: A Cross-Sectional Study. Front. Aging Neurosci..

[80] Tu W.J., Zhao S.J., Xu D.J., Chen H. (2014). Serum 25-hydroxyvitamin D predicts the short-term outcomes of Chinese patients with acute ischaemic stroke. Clin. Sci..

[81] Wajda J., Świat M., Owczarek A.J., Brzozowska A., Olszanecka-Glinianowicz M., Chudek J. (2019). Severity of Vitamin D Deficiency Predicts Mortality in Ischemic Stroke Patients. Dis. Markers.

[82] Wei Z.N., Kuang J.G. (2018). Vitamin D deficiency in relation to the poor functional outcomes in nondiabetic patients with ischemic stroke. Biosci. Rep..

[83] Suthar O.P., Mathur S., Gupta V., Agarwal H., Mathur A., Singh P., Sharma S.L. (2018). Study of Correlation of Serum Vitamin D Levels with Arterial Stiffness and Cardiovascular Morbidity in Elderly Individuals of Western Rajasthan. J. Assoc. Physicians India.

[84] Feng C., Tang N., Huang H., Zhang G., Qi X., Shi F. (2019). 25-Hydroxy vitamin D level is associated with total MRI burden of cerebral small vessel disease in ischemic stroke patients. Int. J. Neurosci..

[85] Michos E.D., Melamed M.L. (2008). Vitamin D and cardiovascular disease risk. Curr. Opin. Clin. Nutr. Metab. Care.

[86] Kashefiolasl S., Leisegang M.S., Helfinger V., Schürmann C., Pflüger-Müller B., Randriamboavonjy V., Vasconez A.E., Carmeliet G., Badenhoop K., Hintereder G. (2021). Vitamin D-A New Perspective in Treatment of Cerebral Vasospasm. Neurosurgery.

[87] Turetsky A., Goddeau R.P., Henninger N. (2015). Low Serum Vitamin D Is Independently Associated with Larger Lesion Volumes after Ischemic Stroke. J. Stroke Cerebrovasc. Dis..

[88] Nie Z., Ji X.C., Wang J., Zhang H.X. (2017). Serum levels of 25-hydroxyvitamin D predicts infarct volume and mortality in ischemic stroke patients. J. Neuroimmunol..

[89] Huang H., Zheng T., Wang S., Wei L., Wang Q., Sun Z. (2016). Serum 25-hydroxyvitamin D predicts early recurrent stroke in ischemic stroke patients. Nutr. Metab. Cardiovasc. Dis..

[90] Lasek-Bal A., Jedrzejowska-Szypulka H., Student S., Warsz-Wianecka A., Zareba K., Puz P., Bal W., Pawletko K., Lewin-Kowalik J. (2019). The importance of selected markers of inflammation and blood-brain barrier damage for short-term ischemic stroke prognosis. J. Physiol. Pharmacol..

[91] Di Napoli M., Singh P. (2009). Is plasma fibrinogen useful in evaluating ischemic stroke patients? Why, how, and when. Stroke.

[92] Rasyid A., Harris S., Kurniawan M., Mesiano T., Hidayat R. (2019). Fibrinogen and LDL Influence on Blood Viscosity and Out-come of Acute Ischemic Stroke Patients in Indonesia. Ann. Neurosci..

[93] Tao L., ShiChuan W., DeTai Z., Lihua H. (2020). Evaluation of lipoprotein-associated phospholipase A2, serum amyloid A, and fibrinogen as diagnostic biomarkers for patients with acute cerebral infarction. J. Clin. Lab. Anal..

[94] Xiao M., Xiao Z.J., Yang B., Lan Z., Fang F. (2020). Blood-Brain Barrier: More Contributor to Disruption of Central Nervous System Homeostasis Than Victim in Neurological Disorders. Front. Neurosci..

[95] Belcher J.D., Chen C., Nguyen J., Milbauer L., Abdulla F., Alayash A.I., Smith A., Nath K.A., Hebbel R.P., Vercellotti G.M. (2014). Heme triggers TLR4 signaling leading to endothelial cell activation and vaso-occlusion in murine sickle cell disease. Blood.

[96] Judd S.E., Morgan C.J., Panwar B., Howard V.J., Wadley V.G., Jenny N.S., Kissela B.M., Gutiérrez O.M. (2016). Vitamin D de-ficiency and incident stroke risk in community-living black and white adults. Int. J. Stroke.

[97] D’Hellencourt C.L., Montero-Menei C.N., Bernard R., Couez D. (2003). Vitamin D3 inhibits proinflammatory cytokines and nitric oxide production by the EOC13 microglial cell line. J. Neurosci. Res..

[98] Zielińska-Nowak E., Cichon N., Saluk-Bijak J., Bijak M., Miller E. (2021). Nutritional Supplements and Neuroprotective Diets and Their Potential Clinical Significance in Post-Stroke Rehabilitation. Nutrients.

[99] Yang F.Z., Jehu D.A.M., Ouyang H., Lam F.M.H., Pang M.Y.C. (2020). The impact of stroke on bone properties and muscle-bone relationship: A systematic review and meta-analysis. Osteoporos. Int..

[100] Carda S., Cisari C., Invernizzi M., Bevilacqua M. (2009). Osteoporosis after stroke: A review of the causes and potential treatments. Cerebrovasc. Dis..

[101] Montiel-Castro A.J., González-Cervantes R.M., Bravo-Ruiseco G., Pacheco-López G. (2013). The microbiota-gut-brain axis: Neurobehavioral correlates, health and sociality. Front. Integr. Neurosci..

[102] Srikantha P., Mohajeri M.H. (2019). The Possible Role of the Microbiota-Gut-Brain-Axis in Autism Spectrum Disorder. Int. J. Mol. Sci..

[103] Levy M., Kolodziejczyk A.A., Thaiss C.A., Elinav E. (2017). Dysbiosis and the immune system. Nat. Rev. Immunol..

[104] Battaglini D., Pimentel-Coelho P.M., Robba C., dos Santos C.C., Cruz F.F., Pelosi P., Rocco P.R.M. (2020). Gut Microbiota in Acute Ischemic Stroke: From Pathophysiology to Therapeutic Implications. Front. Neurol..

[105] Yamashiro K., Kurita N., Urabe T., Hattori N. (2021). Role of the Gut Microbiota in Stroke Pathogenesis and Potential Therapeutic Implications. Ann. Nutr. Metab..

[106] Singh P., Rawat A., Alwakeel M., Sharif E., Al Khodor S. (2020). The potential role of vitamin D supplementation as a gut mi-crobiota modifier in healthy individuals. Sci. Rep..

[107] Charoenngam N., Shirvani A., Kalajian T.A., Song A., Holick M.F. (2020). The Effect of Various Doses of Oral Vitamin D(3) Supplementation on Gut Microbiota in Healthy Adults: A Randomized, Double-blinded, Dose-response Study. Anticancer Res..

[108] Kanhere M., He J., Chassaing B., Ziegler T.R., Alvarez J.A., Ivie E.A., Hao L., Hanfelt J., Gewirtz A.T., Tangpricha V. (2018). Bolus Weekly Vitamin D3 Supplementation Impacts Gut and Airway Microbiota in Adults With Cystic Fibrosis: A Dou-ble-Blind, Randomized, Placebo-Controlled Clinical Trial. J. Clin. Endocrinol. Metab..

[109] Cantarel B.L., Waubant E., Chehoud C., Kuczynski J., DeSantis T.Z., Warrington J., Venkatesan A., Fraser C.M., Mowry E.M. (2015). Gut Microbiota in Multiple Sclerosis. J. Investig. Med..

[110] Amadi C.N., Orish C.N., Frazzoli C., Orisakwe O.E. (2022). Dietary interventions for autism spectrum disorder: An updated systematic review of human studies. Psychiatriki.

[111] Ogbu D., Xia E., Sun J. (2020). Gut instincts: Vitamin D/vitamin D receptor and microbiome in neurodevelopment disorders. Open Biol..

[112] Hussein H.M., Elyamany M.F., Rashed L.A., Sallam N.A. (2022). Vitamin D mitigates diabetes-associated metabolic and cognitive dysfunction by modulating gut microbiota and colonic cannabinoid receptor 1. Eur. J. Pharm. Sci..

[113] Akimbekov N.S., Digel I., Sherelkhan D.K., Lutfor A.B., Razzaque M.S. (2020). Vitamin D and the Host-Gut Microbiome: A Brief Overview. Acta Histochem. Cytochem..

[114] Krutzik S.R., Hewison M., Liu P.T., Robles J.A., Stenger S., Adams J.S., Modlin R.L. (2008). IL-15 links TLR2/1-induced mac-rophage differentiation to the vitamin D-dependent antimicrobial pathway. J. Immunol..

[115] Kempker J.A., Han J.E., Tangpricha V., Ziegler T.R., Martin G.S. (2012). Vitamin D and sepsis: An emerging relationship. Derm.-Endocrinol..

[116] Zhang Y.G., Wu S., Sun J. (2013). Vitamin D, Vitamin D Receptor, and Tissue Barriers. Tissue Barriers.

[117] Gupta A., Prabhakar S., Modi M., Bhadada S.K., Kalaivani M., Lal V., Khurana D. (2016). Effect of Vitamin D and calcium supplementation on ischaemic stroke outcome: A randomised controlled open-label trial. Int. J. Clin. Pract..

[118] Sari A., Durmus B., Karaman C.A., Ogut E., Aktas I. (2018). A randomized, double-blind study to assess if vitamin D treatment affects the outcomes of rehabilitation and balance in hemiplegic patients. J. Phys. Ther. Sci..

[119] Narasimhan S., Balasubramanian P. (2017). Role of Vitamin D in the Outcome of Ischemic Stroke—A Randomized Controlled Trial. J. Clin. Diagn. Res..

[120] Torrisi M., Bonanno L., Formica C., Arcadi F.A., Cardile D., Cimino V., Bramanti P., Morini E. (2021). The role of rehabilitation and vitamin D supplementation on motor and psychological outcomes in poststroke patients. Medicine.

[121] Karasu A.U., Karataş G.K. (2021). Effect of vitamin D supplementation on lower extremity motor function and ambulation in stroke patients. Turk. J. Med. Sci..

[122] Schilling S. (2012). Epidemic vitamin D deficiency among patients in an elderly care rehabilitation facility. Dtsch. Arztebl. Int..

[123] Neo J.J., Kong K.H. (2016). Prevalence of Vitamin D Deficiency in Elderly Patients Admitted to an Inpatient Rehabilitation Unit in Tropical Singapore. Rehabil. Res. Pract..

[124] Bakradze E., McCullough L., Staff I., Nouh A. (2016). Vitamin D deficiency correlates with stroke severity on presentation in in-tracerebral hemorrhage. Stroke.

[125] Türkanoğlu Özçelik A., Öner T., Can Demirdöğen B., Bek V.S., Demirkaya S., Adalı O. (2018). Genetic polymorphisms of vit-amin D3 metabolizing CYP24A1 and CYP2R1 enzymes in Turkish patients with ischemic stroke. Neurol. Res..

